# Intake and Sources of Dietary Fiber, Inflammation, and Cardiovascular Disease in Older US Adults

**DOI:** 10.1001/jamanetworkopen.2022.5012

**Published:** 2022-03-31

**Authors:** Rupak Shivakoti, Mary L. Biggs, Luc Djoussé, Peter Jon Durda, Jorge R. Kizer, Bruce Psaty, Alex P. Reiner, Russell P. Tracy, David Siscovick, Kenneth J. Mukamal

**Affiliations:** 1 Department of Epidemiology, Columbia University Mailman School of Public Health, New York, New York; 2 Department of Biostatistics, University of Washington, Seattle; 3 Division of Aging, Brigham and Women's Hospital, Department of Medicine, Harvard Medical School, Boston, Massachusetts; 4 Boston Veterans Healthcare, Boston, Massachusetts; 5 Department of Pathology and Laboratory Medicine, Larner College of Medicine at the University of Vermont, Burlington; 6 Cardiology Section, San Francisco Veterans Affairs Health Care System, San Francisco, California; 7 Departments of Medicine, Epidemiology and Biostatistics, University of California–San Francisco, San Francisco; 8 Cardiovascular Health Research Unit, Departments of Medicine, Epidemiology, and Health Services, University of Washington, Seattle; 9 Kaiser Permanente Washington Health Research Institute, Seattle; 10 Department of Epidemiology, University of Washington, Seattle; 11 New York Academy of Medicine, New York; 12 Department of Medicine, Beth Israel Deaconess Medical Center, Boston, Massachusetts; 13 Department of Nutrition, Harvard Chan School of Public Health, Boston, Massachusetts

## Abstract

**Importance:**

Higher intake of dietary fiber has been associated with lower inflammation, but whether there are differences in this association by source of dietary fiber (ie, cereal, vegetable, or fruit) has not been studied to date.

**Objectives:**

To evaluate the associations of total fiber intake and source (ie, cereal, vegetable, and fruit fiber intake) with inflammation and to evaluate whether inflammation mediates the inverse association between dietary fiber intake and cardiovascular disease (CVD).

**Design, Setting, and Participants:**

At the baseline visit (1989-1990) of 4125 adults aged 65 years or older in an ongoing US cohort study, dietary intake was assessed by a food frequency questionnaire among study participants without prevalent CVD (stroke and myocardial infarction) at enrollment. Inflammation was assessed from blood samples collected at baseline with immunoassays for markers of inflammation. Multivariable linear regression models tested the association of dietary fiber intake with inflammation. Also assessed was whether each inflammatory marker and its composite derived from principal component analysis mediated the association of baseline cereal fiber intake with development of CVD (stroke, myocardial infarction, and atherosclerotic cardiovascular death) through June 2015. Data from June 1, 1989, through June 30, 2015, were analyzed.

**Exposures:**

Total fiber intake and sources of fiber (cereal, vegetable, and fruit).

**Main Outcomes and Measures:**

Systemic markers of inflammation. Cardiovascular disease was the outcome in the mediation analysis.

**Results:**

Of 4125 individuals, 0.1% (n = 3) were Asian or Pacific Islander, 4.4% (n = 183) were Black, 0.3% (n = 12) were Native American, 95.0% (n = 3918) were White, and 0.2% (n = 9) were classified as other. Among these 4125 individuals (2473 women [60%]; mean [SD] age, 72.6 [5.5] years; 183 Black individuals [4.4%]; and 3942 individuals of other races and ethnicitites [95.6%] [ie, race and ethnicity other than Black, self-classified by participant]), an increase in total fiber intake of 5 g/d was associated with significantly lower concentrations of C-reactive protein (adjusted mean difference, −0.05 SD; 95% CI, −0.08 to −0.01 SD; *P* = .007) and interleukin 1 receptor antagonist (adjusted mean difference, −0.04 SD; 95% CI, −0.07 to −0.01 SD; *P* < .02) but with higher concentrations of soluble CD163 (adjusted mean difference, 0.05 SD; 95% CI, 0.02-0.09 SD; *P* = .005). Among fiber sources, only cereal fiber was consistently associated with lower inflammation. Similarly, cereal fiber intake was associated with lower CVD incidence (adjusted hazard ratio, 0.90; 95% CI, 0.81-1.00; 1941 incident cases). The proportion of the observed association of cereal fiber with CVD mediated by inflammatory markers ranged from 1.5% for interleukin 18 to 14.2% for C-reactive protein, and 16.1% for their primary principal component.

**Conclusions and Relevance:**

Results of this study suggest that cereal fiber intake was associated with lower levels of various inflammatory markers and lower risk of CVD and that inflammation mediated approximately one-sixth of the association between cereal fiber intake and CVD.

## Key Points

**Question:**

What are the associations of total fiber and fiber source with inflammation, and does inflammation mediate the inverse association between cereal fiber intake and cardiovascular disease?

**Findings:**

In this cohort study of 4125 patients, higher total fiber and cereal fiber intakes were associated with lower inflammation. Inflammation mediated approximately one-sixth of the observed inverse association of cereal fiber with cardiovascular disease.

**Meaning:**

Inflammation may play a modest role in mediating the observed inverse association between cereal fiber intake and cardiovascular disease; nevertheless, cereal fiber may have a role in attempts to reduce systemic inflammation, which will need to be tested in future studies.

## Teaser

This cohort study evaluates the association between total fiber and fiber source and inflammation in older patients from the Cardiovascular Health Study.

## Introduction

Low-grade systemic inflammation is associated with an increased risk of various diseases, including cardiovascular disease (CVD) and cancer.^1,2,3^ Thus, modifiable factors that can reduce inflammation may potentially modulate disease risk. One possible modifiable factor is dietary fiber intake, in which higher intakes have been associated with lower systemic inflammation in various studies of healthy adults^4,5^ and adults with specific health conditions (eg, chronic kidney disease,^5^ diabetes,^6^ and obesity^7^).

However, limited data on the association between fiber and inflammation exist for older adults, who have higher levels of inflammation compared with younger adults.^8^ Furthermore, data are lacking on whether the source of fiber (ie, cereal, vegetable, and fruit fiber intake) affects inflammation differently in healthy populations. In addition, most studies of dietary fiber and inflammation have focused on circulating concentrations of C-reactive protein (CRP), an acute phase protein, along with the inflammatory cytokines interleukin 6 (IL-6) and tumor necrosis factor α (TNFα). As a result, further investigation of the association between dietary fiber and systemic inflammation based on source of fiber and study population (eg, healthy elderly individuals) and with diverse markers of inflammation that reflect different aspects of immunity (eg, inflammasome activation, monocyte activation, and other general inflammatory cytokines) is warranted. This information would be useful for the selection of interventions that seek to target specific aspects of inflammation.

The association between dietary fiber and inflammation is of specific interest to the CVD field because higher intakes of dietary fiber, specifically cereal fiber, have been associated with a lower CVD risk.^9,10,11^ A common hypothesized mechanism is that higher intakes of dietary fiber reduce inflammation, which then reduces CVD risk^12,13,14^; multiple studies have shown a positive association between inflammatory markers and development of CVD.^1,2,15,16,17,18^ However, to our knowledge, formal mediation analysis testing whether and to what extent inflammation mediates the observed inverse association between high dietary fiber intake and CVD risk is lacking. Moreover, one particular source of dietary fiber (eg, vegetable fiber) but not another (eg, fruit fiber) could mediate this association; understanding this could inform potential interventions for CVD risk reduction.

To address these research gaps, we studied participants from the Cardiovascular Health Study (CHS), a cohort of adults aged 65 years or older in the United States. We assessed whether total and source-specific dietary fiber intakes were associated with circulating levels of markers of acute-phase inflammation (CRP), inflammasome activation (IL-18 and IL-1 receptor antagonist [IL-1RA]), monocyte or macrophage activation (soluble CD14 and soluble CD163 [sCD163]), and other general inflammatory cytokines (IL-6, soluble IL-2 receptor α, and soluble TNF receptor 1^1,15,19,20^), as well as with incident CVD.^9^

## Methods

### Study Design and Population

This cohort study used data from the CHS, which includes US adults aged 65 years or older. Detailed CHS study procedures have been described elsewhere.^21^ For this analysis of baseline dietary intake and systemic inflammation, we analyzed data from 4125 CHS study participants enrolled from 1989 to 1990 (eMethods in the Supplement). Data analysis was conducted with the categories of Black race and other races and ethnicities. This study was approved by the institutional review boards at the 4 study sites (University of California, Davis; Johns Hopkins University; Wake Forest University; and University of Pittsburgh), and written informed consent was obtained from study participants. The Strengthening the Reporting of Observational Studies in Epidemiology (STROBE) reporting guideline for cohort studies was followed.

### Dietary Intake Assessment

For assessment of long-term usual dietary intake, a food frequency questionnaire was administered to the study participants at the baseline visit. As detailed previously,^9^ this was a 99-item, picture-sort version of the National Cancer Institute food frequency questionnaire^22^ and was validated in a subset of CHS participants (eMethods in the Supplement). Estimates of total energy, total fiber intake, and source of fiber intake (ie, cereal, fruit, and vegetable fiber) were calculated according to the food frequency questionnaire responses and linked nutrient databases.^9,22^ We adjusted fiber intakes for total energy intakes by using the residual method.^23^

### Laboratory Procedures

Fasting blood samples were collected from study participants at the baseline visit. Different circulating markers of inflammation were measured as part of different CHS analyses and substudies.^1,15,24,25,26^ Immunoassays, as previously described^1,15,24,25,26^ and detailed in the eMethods in the Supplement, were used to measure CRP, IL-6, soluble CD14, sCD163, soluble IL-2 receptor α, IL-1RA, IL-18, and soluble TNF receptor 1.

### Follow-up Visits and CVD Events

Follow-up visits (alternating in-person visits and telephone calls) were scheduled every 6 months from baseline through 1999, with information collected on incident diagnoses. After 1999, telephone calls continued every 6 months. Sources for obtaining data on potential cardiovascular events are described in the eMethods in the Supplement. For the purposes of the mediation analysis, the outcome was incident CVD classified as stroke, myocardial infarction, or atherosclerotic cardiovascular death.^9,21^ Follow-up for adjudicated events was completed through June 2015.

### Statistical Analysis

To understand differences in baseline study population characteristics by dietary fiber intake, we categorized study participants by quintile of total fiber intake (grams per day). Multivariable linear regression models were used to assess the association of fiber intake (total, cereal, vegetable, and fruit) with 8 markers of inflammation. The regression coefficients and 95% CIs presented indicate a per-SD increase in log (inflammatory marker) per fiber increase of 5 g/d (ie, SD of total fiber intake). The multivariable model 1 was adjusted for age, sex, race and ethnicity (Black or other [ie, race and ethnicity other than Black, self-classified by participant]), study site, baseline body mass index (BMI), and intake of other fiber types (except for in the total fiber model). The multivariable model 2 was further adjusted for smoking status, physical activity, alcohol consumption, education, protein intake, saturated fat intake, and ratio of polyunsaturated to saturated fat intake in addition to the variables in model 1. The multivariable model 3 was further adjusted for baseline diabetes, blood pressure (systolic and diastolic), lipid levels (triglycerides, low-density lipoprotein cholesterol, and high-density lipoprotein cholesterol), and prevalent heart failure.

To test whether inflammation mediated the association of dietary fiber intake with incident CVD, we first conducted multivariable Cox regression analysis to confirm the association of fiber intake (ie, total, cereal, vegetable, and fruit fiber) with incident CVD previously reported in a shorter follow-up.^9^ The multivariable models (ie, models 1-3) were adjusted for the same variables contained in the linear regression models.

Given the inverse association between cereal fiber intake and CVD, we next tested whether inflammatory markers (log transformed) mediated the association between cereal fiber intake (per an increase of 5 g/d) and incident CVD. For this analysis, we assessed the mediating role of each inflammatory marker separately (except for soluble IL-2 receptor α and sCD163 owing to their smaller numbers), as well as a principal component (PC1) based on the 6 markers. For the PC1, we conducted a standardized principal components analysis and used PC1 as the mediating variable in the analysis. Principal component 1 explained 39% of the variability in the inflammatory markers, with the highest loadings (0.44-0.46) on CRP, IL-6, IL-1RA, and soluble TNF receptor 1. We used the VanderWeele counterfactual-framework approach,^27,28^ adjusting for age, sex, race and ethnicity (Black or other), study site, intakes of other fiber types (fruit and vegetable), protein intake, saturated fat intake, ratio of polyunsaturated to saturated fat, and baseline variables, including BMI, smoking status, physical activity, alcohol consumption, and education. This approach accommodates the interaction of the primary exposure with the mediator of interest. Two-sided *P* values at a significance level of .05 were calculated with linear regression for continuous measures; a nonparametric test for trend across ordered groups for binary measures; and the χ^2^ test for categorical measures with more than 2 categories. All analyses were conducted in Stata version 14.2 (StataCorp LLC) or SAS version 9.4 (SAS Institute), from August 26, 2020, to February 1, 2022.

## Results

### Fiber Intake and Study Population Characteristics

Of 4125 individuals, 0.1% (n = 3) were Asian or Pacific Islander, 4.4% (n = 183) were Black, 0.3% (n = 12) were Native American, 95.0% (n = 3918) were White, and 0.2% (n = 9) were classifed as other. Among the 4125 eligible participants (1652 men [40%]; 2473 women [60%]; mean [SD] age, 72.6 [5.5] years; 183 Black individuals [4.4%]; and 3942 individuals of other races and ethnicities [95.6%]), the mean (SD) energy-adjusted total fiber intake was 16.3 (5.3) g/d. The mean (SD) energy-adjusted cereal fiber intake was 4.2 (2.6) g/d, the mean (SD) vegetable fiber intake was 6.9 (3.3) g/d, and the mean (SD) fruit fiber intake was 5.2 (2.7) g/d.

We next assessed whether baseline study population characteristics were different by quintiles of energy-adjusted total fiber intake (825 individuals in each quintile) (Table 1). Individuals with intakes in the lower quartiles of total fiber were more likely to be men (485 [58.8%]), to be Black (48 [5.8%]), to have less than a high school education (285 [34.5%]), to be current smokers (155 [18.8%]) or former smokers (365 [44.2%]), to drink more alcohol (mean [SD], 4.1 [8.7] beverages/wk), and to engage in less physical activity (mean [SD], 1741.8 [2217.2] kcal/wk) (Table 1). As might be expected, individuals with intakes in the lowest quartiles of total fiber intake also had lower intakes of vegetables (mean [SD], 1.5 [0.9] servings/d) and fruits (mean [SD], 1.3 [0.7] servings/d) (Table 1). Body mass index, blood pressure, antihypertensive medications, and lipid and glucose profiles were similar between quintiles of total fiber intake (Table 1).

**Table 1.** Characteristics of Cardiovascular Health Study Participants at Baseline by Total Fiber Intake (N = 4125)

Characteristic	Total fiber intake, No. (%) of participants					*P* value
	≤11.5 g/d	>11.5-14.5 g/d	>14.5-17.5 g/d	>17.5-21.1 g/d	>21.1 g/d	
No. of participants	825	825	825	825	825	
Age, mean (SD), y	72.6 (5.7)	72.4 (5.4)	72.6 (5.6)	72.7 (5.4)	72.8 (5.7)	.38
Men	485 (58.8)	375 (45.5)	319 (38.7)	249 (30.2)	224 (27.2)	<.001
Women	340 (41.2)	450 (54.5)	506 (61.3)	576 (69.8)	601 (72.8)	
Black participants^a^	48 (5.8)	33 (4.0)	38 (4.6)	28 (3.4)	36 (4.4)	.12
Participants of other races and ethnicities^a^	777 (94.2)	792 (96.0)	787 (95.4)	797 (96.6)	789 (95.6)	
Educational attainment						
<HS	285 (34.5)	245 (29.7)	207 (25.1)	189 (22.9)	171 (20.7)	<.001
HS	238 (28.8)	226 (27.4)	230 (27.9)	254 (30.8)	238 (28.8)	
>HS	302 (36.6)	354 (42.9)	388 (47.0)	382 (46.3)	416 (50.4)	
BMI, mean (SD)	26.4 (4.3)	26.6 (4.5)	26.6 (4.6)	26.3 (4.7)	26.2 (4.7)	.17
BP, mean (SD), mm Hg						
Systolic	135.3 (20.5)	137.0 (22.0)	136.4 (22.0)	134.7 (20.8)	134.9 (21.2)	.17
Diastolic	71.1 (11.4)	71.3 (11.3)	70.5 (11.4)	69.3 (10.6)	69.2 (11.0)	<.001
LDL, mean (SD), mg/dL	129.1 (35.3)	129.9 (35.2)	128.1 (37.9)	130.5 (36.5)	131.0 (35.8)	.26
HDL, mean (SD), mg/dL	52.6 (15.6)	54.4 (15.5)	54.9 (16.3)	55.1 (15.2)	56.0 (15.9)	<.001
Triglycerides, mean (SD), mg/dL	142.1 (81.6)	141.6 (72.6)	139.4 (72.9)	141.1 (70.4)	140.8 (75.9)	.69
Fasting glucose, mean (SD), mg/dL	109.2 (30.3)	110.0 (31.4)	109.2 (30.4)	108.8 (31.3)	108.9 (40.3)	.60
Smoking status						
Never	305 (37.0)	374 (45.3)	400 (48.5)	417 (50.5)	470 (57.0)	<.001
Former	365 (44.2)	347 (42.1)	342 (41.5)	329 (39.9)	300 (36.4)	
Current	155 (18.8)	104 (12.6)	83 (10.1)	79 (9.6)	55 (6.7)	
Alcoholic beverages/wk, mean (SD)	4.1 (8.7)	3.3 (7.1)	2.4 (5.6)	2.0 (5.2)	1.3 (3.3)	<.001
Physical activity, mean (SD), kcal/wk	1741.8 (2217.2)	1692.5 (1901.5)	1778.3 (2037.5)	1828.8 (2229.2)	2091.3 (2222.5)	<.001
Fruit, mean (SD), servings/d	1.3 (0.7)	1.7 (0.8)	2.1 (0.8)	2.5 (0.9)	3.2 (1.0)	<.001
Vegetables, mean (SD), servings/d	1.5 (0.9)	2.0 (1.0)	2.3 (1.0)	2.8 (1.1)	3.8 (1.6)	<.001
Antihypertensive medication	329 (39.9)	338 (41.0)	324 (39.3)	352 (42.7)	353 (42.8)	.17
Diabetes	117 (14.2)	117 (14.2)	118 (14.3)	117 (14.2)	108 (13.1)	.57
CHF	22 (2.7)	25 (3.0)	16 (1.9)	25 (3.0)	14 (1.7)	.26

Abbreviations: BMI, body mass index (calculated as weight in kilograms divided by height in meters squared); BP, blood pressure; CHF, congestive heart failure; HDL, high-density lipoprotein; HS, high school; LDL, low-density lipoprotein.

SI conversion factor: To convert LDL and HDL to milligrams per liter, multiply by 0.1; to convert triglycerides to millimoles per liter, multiply by 0.0113; and to convert fasting glucose to millimoles per liter, multiply by 0.0555.

^a^ Data analysis was conducted with the categories Black race and other races and ethnicities (ie, race and ethnicity other than Black, self-classified by participant). Of 4125 individuals, 0.1% (n = 3) were Asian or Pacific Islander, 4.4% (n = 183) were Black, 0.3% (n = 12) were Native American, 95.0% (n = 3918) were White, and 0.2% (n = 9) were classified as other.

### Association of Dietary Fiber Intake With Inflammation

In multivariable linear regression models adjusted for age, sex, race and ethnicity, study site, BMI, smoking status, physical activity, alcohol consumption, education, dietary protein intake, saturated fat intake, and ratio of polyunsaturated to saturated fat (ie, multivariable model 2), an increase in total fiber intake of 5 g/d was associated with significantly lower concentrations of CRP (adjusted mean difference, −0.05 SD; 95% CI, −0.08 to −0.01 SD; *P* = .007) and IL-1RA (adjusted mean difference, −0.04 SD; 95% CI, −0.07 to −0.01 SD; *P* = .02) (Figure). An increase in total fiber intake of 5 g/d was associated with significantly higher concentrations of sCD163 (adjusted mean difference, 0.05 SD; 95% CI, 0.02-0.09 SD; *P* = .005) (Figure). Results were similar in simpler models (ie, multivariable model 1) and in models that additionally adjusted for other sociodemographic, behavioral, and clinical characteristics (ie, multivariable 3) (eTable in the Supplement).

**Figure.**
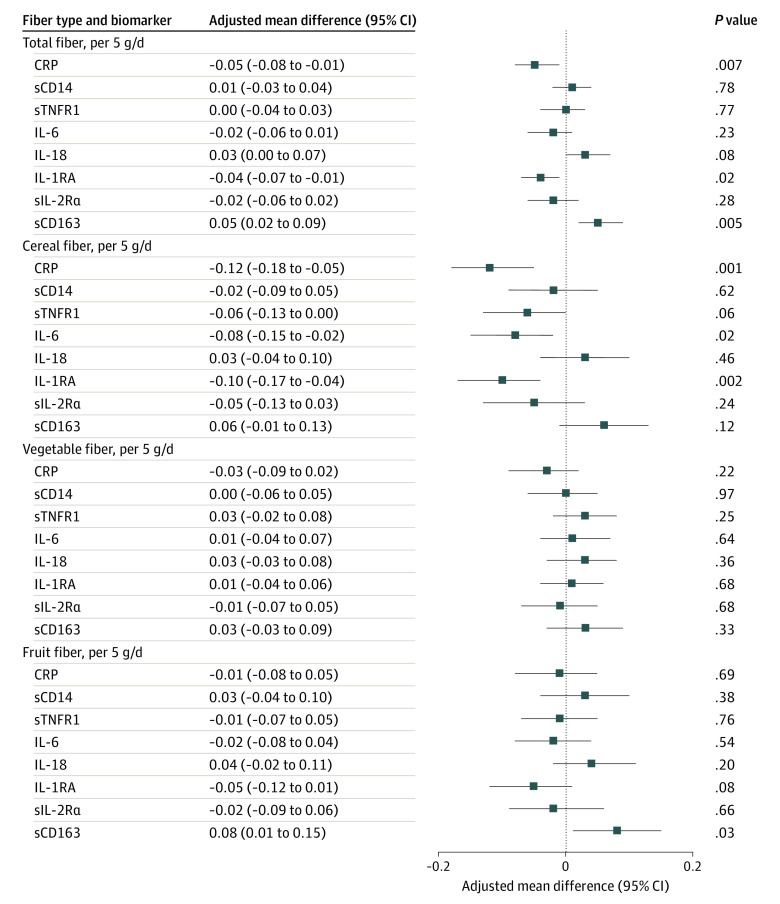
Association of Dietary Fiber With Inflammatory Markers Beta coefficients and 95% CIs are from a linear regression model and represent a per-SD change in log (marker) associated with an increase in fiber of 5 g/d. Results represent data from multivariable model 2, adjusted for age, sex, race and ethnicity, study site, baseline body mass index, other fiber types (except for total fiber model), smoking status, physical activity, alcohol consumption, education, protein intake, saturated fat intake, and ratio of polyunsaturated to saturated fat. CRP indicates C-reactive protein; IL, interleukin; IL-1RA, IL-1 receptor antagonist; sCD14, soluble CD14; sCD163, soluble CD163; sIL-2Rα, soluble IL-2 receptor α; and sTNFR1, soluble TNF receptor 1.

We next assessed these associations by source of fiber (ie, cereal, vegetable, and fruit). An increase in cereal fiber intake of 5 g/d was associated with significantly lower concentrations of CRP (adjusted mean difference, −0.12 SD; 95% CI, −0.18 to −0.05 SD; *P* = .001), IL-6 (adjusted mean difference, −0.08 SD; 95% CI, −0.15 to −0.02 SD; *P* = .02), and IL-1RA (adjusted mean difference, −0.10 SD; 95% CI, −0.17 to −0.04 SD; *P* = .002) in multivariable models (Figure). The multivariable models were adjusted for the same variables as those included in the total fiber intake models, with additional adjustments for other sources of fiber (eg, adjusting for fruit and vegetable fiber in cereal fiber models). However, unlike the total fiber intake, higher levels of cereal fiber intake were not associated with sCD163 concentrations (Figure).

The results for vegetable fiber intake and fruit fiber intake were, however, different compared with those for cereal fiber and total fiber. Higher intakes (ie, per 5 g/d) of vegetable fiber were not significantly associated with any of the markers in multivariable model 2 (Figure). For fruit fiber, higher intakes were associated with significantly higher concentrations of sCD163 (adjusted mean difference, 0.08 SD; 95% CI, 0.01-0.15 SD; *P* = .03) (Figure).

### Inflammation as a Mediator Between Fiber Intake and CVD

During a median follow-up time of 11.9 years (range, 0.01-26.0 years), there were 1941 incident CVD events. In multivariable Cox regression model 1, an increase in total fiber of 5 g/d (hazard ratio [HR], 0.95; 95% CI, 0.91-0.99; *P* = .01) and an increase in cereal fiber of 5 g/d (HR, 0.86; 95% CI, 0.79-0.95; *P* < .001), but not vegetable or fruit fiber, were associated with a lower risk of CVD (Table 2). Similar results (adjusted HR, 0.83; 95% CI, 0.72-0.96) were observed in sensitivity analysis limiting the follow-up time to 10 years. When analyzed by CVD subtype, HR estimates for cereal fiber were similar to those for stroke (adjusted HR, 0.85 [95% CI, 0.72-1.00]; adjusted HR, 0.78 [95% CI, 0.63-0.97] when follow-up was limited to 10 years) but not myocardial infarction and atherosclerotic coronary heart disease death (adjusted HR, 0.94; 95% CI, 0.83-1.07).

**Table 2.** Association of Dietary Fiber With Incident Cardiovascular Disease^a^

Fiber type per 5 g/d	Model 1		Model 2		Model 3	
	HR (95% CI)	*P* value	HR (95% CI)	*P* value	HR (95% CI)	*P* value
Total	0.95 (0.91-0.99)	.01	0.98 (0.93-1.04)	.53	0.98 (0.92-1.03)	.36
Cereal	0.86 (0.79-0.95)	<.001	0.90 (0.81-1.00)	.05	0.92 (0.83-1.02)	.11
Vegetable	0.97 (0.91-1.05)	.48	0.97 (0.90-1.06)	.53	0.96 (0.88-1.04)	.30
Fruit	1.00 (0.91-1.09)	.95	1.06 (0.96-1.17)	.28	1.04 (0.94-1.15)	.42

^a^ Hazard ratios (HRs) and 95% CIs are from a Cox regression model and represent hazards of cardiovascular disease associated with an increase in fiber of 5 g/d. Model 1 was adjusted for age, sex, race and ethnicity, study site, baseline body mass index, and other fiber types (except for total fiber model). Model 2 was adjusted for model 1 covariates plus smoking status, physical activity, alcohol consumption, education, protein intake, saturated fat intake, and ratio of polyunsaturated to saturated fat. Model 3 was adjusted for model 2 covariates plus baseline diabetes, systolic blood pressure, diastolic blood pressure, low-density lipoprotein, high-density lipoprotein, triglycerides, and heart failure.

Next, we conducted mediation analysis to examine whether and to what extent the specific inflammatory markers mediated the association between high cereal fiber intake and lower CVD. Table 3 shows the HRs and 95% CIs of the total effect, which is the effect of cereal fiber on CVD; the natural direct effect, which is the effect of cereal fiber on CVD that is not mediated by inflammatory markers; and the natural indirect effect, which is the effect of cereal fiber on CVD that is mediated by inflammatory markers. In general, the natural direct effect greatly exceeded the indirect effect, although the latter was significant for several markers. The percentage mediated by individual inflammatory markers ranged from 1.5% for IL-18 to 14.2% for CRP (Table 3). To account for correlations between inflammatory markers, we also conducted principal components analysis of these markers. When PC1 was used as the mediating variable in the analysis, the natural indirect effect was modest but significantly inverse (HR, 0.97; 95% CI, 0.95-0.99), and PC1 mediated 16.1% of the effect of cereal fiber on CVD (Table 3).

**Table 3.** Association Between Cereal Fiber and Incident Cardiovascular Disease Mediated by Inflammatory Markers^a^

Inflammatory marker^b^	Hazard ratio (95% CI)			% Mediated
	Total association	Natural direct association	Natural indirect association	
IL-6	0.87 (0.78-0.98)	0.88 (0.78-0.98)	0.99 (0.98-0.99)	9.0
CRP	0.88 (0.79-0.99)	0.90 (0.81-1.00)	0.98 (0.97-0.99)	14.2
sTNFR1	0.88 (0.79-0.99)	0.89 (0.80-0.99)	0.99 (0.98-1.00)	7.5
IL-1RA	0.89 (0.80-0.99)	0.91 (0.81-1.01)	0.99 (0.98-0.99)	11.4
IL-18	0.89 (0.80-0.99)	0.89 (0.80-0.99)	1.00 (0.99-1.01)	1.5
sCD14	0.89 (0.80-0.99)	0.89 (0.80-0.99)	0.99 (0.99-1.01)	1.8
PC1	0.84 (0.74-0.96)	0.87 (0.76-0.98)	0.97 (0.95-0.99)	16.1

Abbreviations: CRP, C-reactive protein; IL, interleukin; IL-1RA, IL-1 receptor antagonist; PC1, principal component 1; sCD14, soluble CD14; sTNFR1, soluble tumor necrosis factor receptor 1.

^a^ Model was adjusted for age, sex, race and ethnicity, study site, other fiber types, protein intake, saturated fat intake, ratio of polyunsaturated to saturated fat, baseline body mass index, smoking status, physical activity, alcohol consumption, and education. An interaction term between cereal fiber and inflammation was included in the model. The total effect, natural direct effect, natural indirect effect, and percentage mediated were calculated using the VanderWeele counterfactual-framework approach.^27,28^ The total effect shown is slightly different from that in Table 2 and also slightly different between markers because of differences in sample size for each marker (specified in Table 2).

^b^ Per-SD change in inflammatory marker.

In additional analyses, we considered the role of BMI in the observed associations. If increased cereal fiber intake is associated with reduced adiposity, which in turn reduces inflammation, adjusting for BMI in the models could attenuate the observed mediating effect of inflammation. Removing BMI from the model was associated with a slightly increased mediating role of inflammation, in which the percentage mediated by PC1 was 18.7%.

## Discussion

In this prospective cohort study of older adults, higher intakes of total fiber were associated with lower levels of various inflammatory markers, and this inverse association was primarily due to cereal fiber intake. Vegetable and fruit fiber intakes were not consistently associated with lower levels of inflammatory markers. These results suggest that specifically cereal fibers might be more effective in reducing systemic inflammation, which will need to be tested in interventional studies of specific populations. In addition, cereal fiber was associated with a lower risk of CVD, although inflammation mediated less than 20% of the observed inverse association between cereal fiber and CVD. This suggests that the association of cereal fiber with CVD risk is primarily due to factors (eg, other mediators, a direct role played by cereal fiber, or cereal fibers replacing other unhealthful foods) other than systemic inflammation.

Multiple studies, as well as data from large population-based observational studies, have shown an inverse association between total fiber intakes and general markers of inflammation, including CRP, IL-6, and TNFα, in healthy adults and in those with specific metabolic diseases.^4,5,6,7^ In this study, we confirmed these associations in older adults, who tend to have higher levels of inflammation, and extended the panel to include soluble TNF receptor 1 and soluble IL-2 receptor α, proinflammatory cytokines associated with adverse outcomes in this cohort, including mortality.^15^ More important, we observed that these associations differed by source of fiber, with cereal fiber, but not vegetable or fruit fiber, being a primary component of this association. An earlier study of individuals with diabetes also noted that cereal fiber, but not fruit or vegetable fiber, was associated with lower levels of circulating CRP,^6^ but the results were lacking in healthy populations (including elderly individuals) or other markers.

Given that unresolved inflammation is associated with both an increased risk of multiple diseases and their outcomes,^1,2,3^ studies should investigate the specific mediating effect of inflammation on these outcomes. In instances in which it plays a mediating role, these findings on the source of fiber are important in the context of a potential intervention to reduce systemic inflammation. Our results suggest that the source of fiber is an important factor to consider, with cereal fiber likely to be most effective in reducing inflammation. Although there are data to suggest that fiber in general might have anti-inflammatory effects by improving gut function, modifying diet and satiety (eg, reduced fat and total energy intake), and improving lipid and glucose profile metabolism,^29,30,31,32,33^ why cereal fiber but not vegetable or fruit fiber is associated with lower inflammation is not clear and warrants further investigation.

These findings on source of fiber could partly explain the discrepancy in the results from prebiotic intervention trials that seek to reduce inflammation^34^ because the prebiotics are from different sources. Similarly, differences in the source of fiber intake in specific populations (eg, low cereal fiber intake and high vegetable fiber intake) may also help explain differences in risk for a particular disease affected by inflammation. These findings will need to be studied, including whether there are differences by type of cereal fiber and whether cereal fiber per se or other properties of cereal fiber–rich foods might contribute to lower inflammation.

Our results also showed differences in the association of fiber with inflammatory markers involved in inflammasome activation. An inverse association of total fiber intake and cereal fiber intake with IL-1RA was observed, whereas there was no association of total fiber or any of the sources of fiber with IL-18. IL-1RA is in fact an anti-inflammatory cytokine of the IL-1 family, in which its binding to the IL-1R blocks the binding of proinflammatory IL-1α and IL-1β to IL-1R.^35,36^ Thus, IL-1RA inhibits IL-1β and inflammasome activation. Although we did not measure IL-1β in this study, we hypothesized that fiber is inversely associated with IL-1β levels and that there is an association with IL-1RA because levels of circulating IL1-RA may reflect an anti-inflammatory negative feedback response to levels of IL-1β.^35,36^ The potential reasons for the observed differences in the association of fiber with IL-1RA and IL-18 are likely associated with the differential effect on IL-1 and IL-18 pathways^35^ but warrant further investigation.

The data from our mediation analysis showed that inflammation had only a modest mediating statistical association with the observed inverse association, noted in this analysis and in our earlier analysis from the CHS,^9^ between cereal fiber and CVD. This suggests that cereal fiber is associated with lower CVD risk, and especially stroke risk, largely through mechanisms other than inflammation (or at least the inflammatory pathways studied here). Other ways cereal fiber might reduce CVD risk could be through direct associations or other mediators (eg, glucose metabolism, lipid profile,^37^ or gut microbiome or short-chain fatty acids^38^). The inverse association between dietary fiber and CVD is commonly hypothesized to be mediated through inflammation^12,13,14^; our results provide important clarification of this hypothesis, suggesting that inflammation is likely not a major mediator of the observed association. Moreover, these findings do not preclude a major mediating role of inflammation in other conditions associated with diet, such as diabetes or obesity.

### Strengths and Limitations

The strengths of this study include data from a large and well-characterized prospective cohort of elderly individuals, with detailed data on dietary intake, inflammation, and incidence of CVD. This study confirmed previously observed associations between dietary fiber, inflammation, and CVD and extended those investigations to include the source of the fiber, a larger panel of inflammatory markers, and a rigorous mediation analysis.

This study also has limitations. These include missing information on additional immune markers that could help explain the observed associations (eg, IL-1β), measurement error associated with intake data from food frequency questionnaires, a lack of repeated measures, the possibility of limited generalizability because this study was conducted with older adults, and the potential for residual confounding.

## Conclusions

In conclusion, higher intakes of cereal fiber, but not vegetable or fruit fiber, were associated with lower levels of inflammation in older adults. Cereal fiber may have a role in attempts to reduce systemic inflammation, and this finding will need to be studied further. In addition, inflammation had only a modest role in mediating the observed inverse association between cereal fiber and CVD. This suggests that factors other than inflammation may play a larger role in the cereal fiber–associated reduction in CVD.

## Notes

Supplement. **eMethods.** Data From 4125 Cardiovascular Health Study Participants Enrolled in 1989 to 1990 **eTable.** Association of Dietary Fiber With Inflammatory Markers
