# Supplementary material for: Intake and Sources of Dietary Fiber, Inflammation, and Cardiovascular Disease in Older US Adults
Source: JAMA Netw Open. 2022 Mar 31;5(3):e225012. doi: 10.1001/jamanetworkopen.2022.5012 (PMC8972036; doi:10.1001/jamanetworkopen.2022.5012)
Supplement: Supplement. — eMethods. Data From 4125 Cardiovascular Health Study Participants Enrolled in 1989 to 1990 eTable. Association of Dietary Fiber With Inflammatory Markers [file jamanetwopen-e225012-s001.pdf]

Supplemental Online Content

Shivakoti R, Biggs ML, Djoussé L, et al. Intake and sources of dietary fiber, inflammation, and cardiovascular disease in older US adults. *JAMA Netw Open*. 2022;5(3):e225012. doi:10.1001/jamanetworkopen.2022.5012

eMethods. Data From 4125 Cardiovascular Health Study Participants Enrolled in 1989 to 1990

eTable. Association of Dietary Fiber With Inflammatory Markers

This supplemental material has been provided by the authors to give readers additional information about their work.

© 2022 Shivakoti R et al. *JAMA Network Open.*

eMethods. Data From 4125 Cardiovascular Health Study Participants Enrolled in 1989 to 1990

Study Design and Population:

We utilized data from the CHS study, a cohort of U.S. adults 65 years and older. Detailed study procedures of the CHS study have been described elsewhere[21]. In brief, 5888 study participants were enrolled, based on recruitment from randomly generated Medicare eligibility lists, in 1989-1990 and 1992-1993 at four communities: Sacramento County in California, Forsyth County in North Carolina, Pittsburgh in Pennsylvania and Washington County in Maryland. Individuals that were institutionalized, wheelchair-bound at home, receiving treatment for cancer, not able to consent and expected to move out of their community in the subsequent 3 years were excluded from this study.

At the baseline visit, detailed information was collected on sociodemographic information, clinical history including medications, physical examination, anthropometrics and dietary intake. In addition, laboratory evaluations were performed and biological samples, including plasma were collected and stored for future use.

For this analysis of baseline dietary intake and systemic inflammation, we focused only on individuals enrolled in 1989-1990 (N=5201) since the dietary questionnaire was not administered at baseline for those enrolled in 1992-1993. Furthermore, given our secondary objective to assess the potential mediating role of inflammation in the observed relationship between baseline dietary fiber intake and prospective CVD, we excluded those with known stroke (N=158) and myocardial infarction (MI) (N=515) at baseline. After further excluding those with no dietary data (N=376) or with implausible energy intakes (<500 or >5000 kcal/day; N=27), the sample size for this analysis was 4125. Study population characteristics, including age, sex, BMI and race were similar between individuals with and without missing inflammatory data.

Ethics Approval:

Informed consent was obtained from all study participants and US Department of Health and Human studies guidelines for human studies were followed.

Dietary Intake Assessment:

Relevant to this analysis of dietary fiber, a validation study previously conducted in a subset of CHS participants showed that the FFQ was a valid tool for estimating fiber intake[9]; the energy-adjusted de-attenuated correlation coefficient of log-transformed values of total fiber intake obtained from FFQ and multiple 24-hour recalls was 0.68.

This supplemental material has been provided by the authors to give readers additional information about their work.

© 2022 Shivakoti R et al. *JAMA Network Open.*

Laboratory Procedures:
CRP was measured using a validated in-house high-sensitivity enzyme-linked immunosorbent assay (ELISAs) developed at the CHS core laboratory[1,23]. The sensitivity of the assay was 0.007 mg/L, with an assay range of 0.08-9.0 mg/L. Using single-plex kits from R&D Systems, levels of IL-6 levels (high-sensitivity Quantikine HS Human IL-6 immunoassay)[1], sCD14 (cat # CD140)[1], sCD163[24] and sIL-2Rα[25] were measured. Mesoscale chemiluminiscent assays were used to measure IL-1-RA, IL-18 and sTNFR1[15]. IL-1RA and IL-18 were measured using a human 2-plex kit following manufacturer's directions (Mesoscale Discovery Technology (MSD)[15]. A single-plex assay with a commercially available MSD MULTI-SPOT 96-well human ultrasensitive plates were used to determine levels of sTNFR1[15]. Sensitivity of the assays were 0.6 pg/mL for IL-18, 1.2 pg/mL for IL-1RA and 0.48 pg/mL.

Follow-up Visits and CVD events:
Information on potential cardiovascular events were obtained from various sources including participant report and physician questionnaires, medical records, death certificates and medical examiner forms, along with Centers for Medicare and Medicaid Services hospitalization records and available imaging scans[9,21,26]. CHS has a central committee to adjudicate and classify cardiac and stroke events.

Statistical analysis:

Missing data for fiber measures and inflammatory markers were not imputed. Among participants eligible for the analysis, <2% of covariate data were missing. Missing covariate data were imputed using multiple imputation as described previously (PMID: 12505893). Based on the very small amount of missing data, a single imputation was used.

We also assessed the functional form of the dietary fiber-CVD association using generalized additive models and tested for departures from linearity using the gain statistic. There was no evidence of meaningful non-linearity of any of the fiber-CVD associations (p>0.05 for all).

This supplemental material has been provided by the authors to give readers additional information about their work.

© 2022 Shivakoti R et al. *JAMA Network Open.*

eTable. Association of Dietary Fiber With Inflammatory Markers

		**Model 1**		**Model 3**	
	N	Beta (95% CI)	p-value	Beta (95% CI)	p-value
**Total fiber**					
CRP	4091	-0.07 (-0.10, -0.05)	<0.001	-0.05 (-0.08, -0.01)	0.008
sCD14	3855	-0.02 (-0.05, 0.00)	0.083	0.01 (-0.03, 0.04)	0.656
TNFaR1	3956	-0.04 (-0.06, -0.01)	0.003	-0.00 (-0.04, 0.03)	0.792
IL-6	3785	-0.07 (-0.09, -0.04)	<0.001	-0.02 (-0.06, 0.01)	0.222
IL-18	3955	-0.01 (-0.04, 0.02)	0.450	0.03 (-0.00, 0.07)	0.054
IL-1RA	3955	-0.06 (-0.09, -0.03)	<0.001	-0.04 (-0.07, -0.00)	0.025
sIL-2Ra	3073	-0.04 (-0.07, -0.01)	0.005	-0.02 (-0.06, 0.02)	0.263
sCD163	3621	0.05 (0.02, 0.08)	0.001	0.05 (0.01, 0.09)	0.007
**Cereal fiber**					
CRP	4091	-0.15 (-0.21, -0.09)	<0.001	-0.11 (-0.17, -0.04)	0.002
sCD14	3855	-0.06 (-0.12, 0.00)	0.063	-0.00 (-0.07, 0.07)	0.993
TNFaR1	3956	-0.10 (-0.16, -0.04)	0.001	-0.05 (-0.11, 0.01)	0.128
IL-6	3785	-0.13 (-0.19, -0.07)	<0.001	-0.07 (-0.14, -0.00)	0.044
IL-18	3955	-0.04 (-0.10, 0.02)	0.215	0.04 (-0.03, 0.11)	0.234
IL-1RA	3955	-0.13 (-0.19, -0.07)	<0.001	-0.09 (-0.16, -0.03)	0.003
sIL-2Ra	3073	-0.08 (-0.15, -0.01)	0.018	-0.04 (-0.12, 0.04)	0.333
sCD163	3621	0.04 (-0.03, 0.10)	0.251	0.07 (0.00, 0.14)	0.044
**Vegetable fiber**					
CRP	4091	-0.05 (-0.09, 0.00)	0.052	-0.03 (-0.08, 0.02)	0.281
sCD14	3855	-0.01 (-0.06, 0.03)	0.552	0.00 (-0.05, 0.06)	0.918
TNFaR1	3956	-0.00 (-0.05, 0.04)	0.980	0.04 (-0.01, 0.08)	0.163
IL-6	3785	-0.01 (-0.06, 0.03)	0.584	0.02 (-0.04, 0.07)	0.521
IL-18	3955	-0.01 (-0.06, 0.04)	0.737	0.03 (-0.02, 0.09)	0.251
IL-1RA	3955	-0.01 (-0.06, 0.03)	0.582	0.02 (-0.03, 0.07)	0.474
sIL-2Ra	3073	-0.03 (-0.08, 0.03)	0.293	-0.01 (-0.08, 0.05)	0.635
sCD163	3621	0.03 (-0.02, 0.08)	0.193	0.03 (-0.03, 0.08)	0.340
**Fruit fiber**					
CRP	4091	-0.05 (-0.11, 0.01)	0.080	-0.02 (-0.09, 0.04)	0.449
sCD14	3855	-0.01 (-0.07, 0.05)	0.801	0.02 (-0.05, 0.09)	0.534
TNFaR1	3956	-0.04 (-0.09, 0.02)	0.202	-0.02 (-0.08, 0.04)	0.437
IL-6	3785	-0.09 (-0.15, -0.03)	0.005	-0.04 (-0.10, 0.03)	0.257
IL-18	3955	0.01 (-0.05, 0.07)	0.724	0.03 (-0.03, 0.10)	0.324
IL-1RA	3955	-0.07 (-0.12, -0.01)	0.023	-0.07 (-0.12, -0.01)	0.029
sIL-2Ra	3073	-0.03 (-0.10, 0.04)	0.372	-0.02 (-0.09, 0.05)	0.569
sCD163	3621	0.08 (0.02, 0.15)	0.010	0.06 (-0.01, 0.13)	0.072

Beta-coefficients and 95% confidence intervals (CI) are from a linear regression model and represent a per-standard deviation (SD) change in log(marker) associated with a 5g/d increase in fiber

Model 1 adjusted for age, sex, race, study site, baseline body mass index (BMI), and other fiber types (except for total fiber model)

Model 3 adjusts for Model 1 covariates plus smoking status, physical activity, alcohol consumption and education, protein intake, saturated fat intake and ratio of polyunsaturated/saturated fat, diabetes, systolic BP, diastolic BP, LDL, HDL, triglycerides, and heart failure

This supplemental material has been provided by the authors to give readers additional information about their work.
